# Capturing the HIV-related social exclusion practices experienced by key populations through photovoice: an interpretative phenomenological study

**DOI:** 10.1186/s12978-024-01832-y

**Published:** 2024-07-15

**Authors:** Ami Kamila, Widyawati Widyawati, Mubasysyir Hasanbasri, Mohammad Hakimi

**Affiliations:** 1 Doctoral Program, Faculty of Medicine, Public Health, and Nursing, Universitas Gadjah Mada, Yogyakarta, Indonesia; 2 Faculty of Health Science, Universitas ‘Aisyiyah Bandung, Bandung, West Java Indonesia; 3 Pediatric and Maternity Nursing Department, Faculty of Medicine, Public Health, and Nursing, Universitas Gadjah Mada, Yogyakarta, Indonesia; 4 Department of Biostatistics, Epidemiology, and Population Health, Faculty of Medicine, Public Health, and Nursing, Universitas Gadjah Mada, Yogyakarta, Indonesia; 5 Obstetrics and Gynecology Department, Faculty of Medicine, Public Health, and Nursing, Universitas Gadjah Mada, Yogyakarta, Indonesia

**Keywords:** Photovoice, Key populations, HIV, Social exclusion, Stigma and discrimination

## Abstract

**Background:**

Key populations are defined as groups that are susceptible to HIV, including Men Sex with Men (MSM), Transgender (TG), Persons who Inject Drug (PID), and Female Sex Worker (FSW). These key populations groups are among the fastest-growing populations in Indonesia. These vulnerable groups are ostracized by society and health services, which makes it difficult to get treatment. This project was carried out to investigate the different experiences and perspectives of these key populations in facing and addressing social and spiritual exclusion.

**Methods:**

A qualitative phenomenological study using photovoice was carried out from July to December 2022. Key populations comprising MSM, TG, PID, and FSW were recruited from community-based peer groups in West Bandung Regency using snowball sampling. This was followed by the Photovoice stages, from workshops to focus group discussions and interviews with audio recordings. Furthermore, thematic data analysis was carried out by interpretative participant narratives and photographs supported by Atlas.ti software.

**Result:**

Eighteen participants comprising four MSM, five TG, four PIDs, and five FSWs participated in this research. Among these eighteen participants, six were HIV-negative, including 3 PIDs and 3 FSWs, while the remaining were positive. The analysis of the collected data identified four main themes: 1) limited access like unequal treatment, disadvantage, and harassment, 2) social and spiritual impact, 3) coping mechanisms, and 4) self-reflection through photovoice. These results showed that social exclusion occurred in an environment where community values, beliefs, and norms dehumanised these key populations, and where removal of support and care was prominent. Despite these challenges, participant resilience was evidenced by using internal resources and peer support as coping mechanisms. The participants considered photovoice as a tool to foster self-confidence and self-awareness through a reflective process.

**Conclusions:**

The findings of this study highlight the emphasis on participants' openness in sharing their experiences, which can build empathy and promote a more inclusive community in HIV prevention efforts. This research findings can be used to inform HIV policy and practice and inclusion of these key populations in the community. We advocate making the photovoice efforts accessible to a wider audience through exhibitions and various media.

## Background

HIV-related key populations are those who are at high risk and vulnerable to HIV infection due to their risky sexual behavior. In addition, these key populations also face challenging social environments in their communities. Key populations include men who have sex with men (MSM), transgender (TG), PIDs, and female sex workers (FSW) [1]. Legal and social issues, such as social relationships, social influences, and the environment in which they live, along with stigmatizing attitudes by healthcare workers that create barriers to health service access that further exacerbate their vulnerability to HIV [2–4]. These individuals face multiple stigmas and discrimination associated with HIV status, behaviour, and identity. Furthermore, these challenges have a significant impact on life decisions and access to health services, including HIV prevention [5–7].

Stigma, a practice of social exclusion, is the major obstacle to reducing the incidence of HIV and AIDS in Indonesia [8–10]. HIV stigma has mechanisms that negatively impact people at risk of HIV, with manifestations such as prejudice, stereotypes and discrimination, significantly affecting their behaviour, psychological well-being, social life and health [11–13]. This stigma and discrimination is enacted by health workers, family, and the community, who judge those living with HIV as "dirty and sinful people" [12]. The majority of the population in Indonesia considers it a violation of religious norms to be part of key population communities. Religious leaders have little involvement in HIV prevention programs in Indonesia. The participation of religious leaders in providing education that can increase community knowledge and help the community interpret religious beliefs through health promotion is still minimal. The minimum involvement of religious leaders has led to high rates of stigma and discrimination in key populations [14]. These factors are a lack of knowledge about how HIV is transmitted, fear of contracting HIV, and negative social and moral perceptions of People Living with HIV (PLHIV) that trigger and encourage stigma and discrimination [13]. As a result, they tend to hide their HIV status and risk behaviour and form hidden communities, making the spread of HIV infection challenging to control [15, 16].

Negative perceptions of key populations increase societal stigma and discrimination [17]. In addition, the absence of positive images in the media can generate empathy and foster a more inclusive and supportive society [18]. Therefore, the media needs to support programs to reduce HIV stigma and discrimination actively. These programs include engaging key populations and presenting objective content about individuals' lives [19].

Using the photovoice technique, key populations can actively participate in creating media narratives based on authentic stories. The photos taken by these individuals serve as expressive symbols, depicting feelings and experiences in response to prevalent practices of social exclusion and rights issues across various aspects of social life. With its creative approach, Photovoice has proven effective in informing certain events that drive social change. It has also successfully reduced stigma and discrimination while fostering a climate that encourages the target populations to share experiences more openly [20–25].

Previous research has used photovoice as a strategy to overcome stigma, addressing not only HIV/AIDS but also extending to cases such as mental illness, drug use, and sexual minority groups. These investigations focus on empowering stigmatized individuals to express their feelings through photographs, both in societal and familial contexts [21, 22, 26, 27]. However, this research has not conveyed this empowering message through educational media formats like photo booklets. Educational media is a powerful tool for promoting sustainable social change, offering the advantage of broader dissemination through an extensive network beyond the limitations of exhibitions.

This research aims to clarify real-life experiences and convey essential messages through photovoice. The main objective is to contribute to the establishment of an inclusive environment.

## Methods

### Study design

A phenomenological approach was adopted in this study to explore the emotional dimensions, perceptions, and personal meanings and to gain an in-depth understanding of the perspectives and experiences of the key populations [28]. Within this phenomenological approach we have used the photovoice method, helping to detail complex layers of experience that may not be revealed through other research approaches.

Photovoice, a community-based participatory documentary photography method, provides a flexible approach designed to suit the research needs [29]. Developed by Wang in the 1990s, photovoice has been widely applied in various ways within public health research [23, 30, 31] and proven effective in capturing personal experiences, including prioritizing perspectives among vulnerable populations often overlooked in social discourse [28, 31, 32], supporting individual empowerment [33], and providing opportunities for social action and policy changes that can influence their future lives [23].

The first author has extensive experience in qualitative research with a primary focus on key populations. Efforts made to minimize subjectivity were as follows: (1) interviews were conducted by research assistants who had experience in conducting data collection with interviews in qualitative research, (2) transcripts of the interviews were prepared with the assistance of https://www.transkrip.id/, and the transcripts were then confirmed with the participants, (3) The research team carried out the analysis of transcript results. Meanwhile, the researcher became a facilitator to ensure the research followed the protocol.

The data collection process followed the key procedures of the photovoice method: 1) members of key populations were recruited and attended a photography workshop and were asked to take photos that portrayed their experiences of social exclusion, 2) Focus Group Discussions (FGDs), 3) interviews and 4) interpretation of the captured photos.

The SHOWED technique and explanations guided the participants in describing their photographs. This technique comprised a series of questions: 1) What is shown or What can be seen here? 2) What is happening here?; 3) How does this relate to their lives?; 4) How does this concern the existing situation?; 5) How can this image be used to educate individuals?; 6) What should be done about this? [28, 34]. In addition to these structured questions, supplementary research inquiries were used to thoroughly explore the experiential narratives behind the captured photographs of the participants, focusing on their social lives as key populations [21, 35]. Furthermore, this research examined participants' efforts through self-reflections [22] and photovoice [23].

The Focus Group Discussion (FGD) was divided into four groups, each consisting of members from diverse communities within the key populations. A moderator facilitated each group and guided participants in exploring their captured photographs. Guided by a predefined set of questions, which included topics such as the context of the photograph, the emotions it evoked, and its relevance to the research, participants revealed the meanings of their photographs by presenting and discussing these pictures within the respective groups.

After the FGD, face-to-face interviews were conducted to explore experiences related to social exclusion. These interviews aimed to understand the impacts on social and spiritual life and how individuals coped with these experiences using stress-coping mechanisms. Interviews were conducted once on the same day for each participant for 40–60 minutes. This research adhered to the criteria for the qualitative approach established in the Consolidated Criteria for Reporting Qualitative Studies (COREQ) [36].

### Participants and eligibility criteria

Study participants were recruited from key populations (MSM, FSW, and Transgender women) residing in West Bandung Regency, Indonesia, using purposive and snowball sampling techniques. Prior to participant recruitment, the researcher coordinated with West Bandung AIDS Commission and Peer Support Groups to explain the research and data collection procedures, including the eligibility criteria. West Bandung AIDS Commission and Peer Support Groups assisted researchers in recruitment. In addition, the West Bandung AIDS Commission and Peer Support Groups also recruited participants in the PID group by snowball sampling, which was an important sampling method for PID, as members of this key population were more difficult to access and identify directly than other groups. The inclusion criteria required these individuals to reside in West Bandung Regency, have experienced social exclusion, stigma, and discrimination, own specific devices such as a smartphone or digital camera, and be willing to provide written consent at each research stage. Participants who do not participate and have difficulty understanding and following the study protocol will be excluded. The inclusion and exclusion criteria are done to maintain the research data's integrity, validity, and quality. Eighteen eligible participants were selected: four MSM, five TG, four PID, and five FSW.

### Procedure

Prior to participation in the project, all study participants provided informed consent. The data collection process was conducted based on the following four stages of photovoice method: (1) introductory workshop, (2) photography capture, (3) SHOWED Mnemonic and interviews, and (4) interpretation of photographs for exhibition and photo booklet purposes, organized at different times.

A three-hour workshop was conducted in the initial stage of the research, facilitated by a professional journalism photographer lecturer. This first stage, conducted before participants engaged in photography, aimed to guide the individuals on the photovoice concept, photography techniques using specific devices, including smartphones and digital cameras, photography ethics, and personal safety. All participants convened during this first stage and shared stories about their experiences and situations, fostering trust and group cohesion among group members.

In the second stage, participants had two weeks for photography capture. Whilst participants were able to take as many photos as they wanted, they were also instructed to select three to five photographs that best illustrated their experiences, perspectives, and emotions of HIV exclusion. These captured photographs served as visual representations and mediums to convey messages about the challenges encountered in daily life while addressing social exclusion, stigma, and discrimination.

The third stage used SHOWED Mnemonic [28] to interpret the photographs through Focus Group Discussions (FGD) and interviews. This technique assisted participants to clarify the narratives behind each captured photograph, particularly those narratives offering in-depth explanations of the background and personal significance of their photo and story. Discussions and interviews were audio-recorded and transcribed for data analysis using the Atlas.ti software package. Field notes captured during FGDs and interviews were actively observed, and certain events or elements not captured by the audio recorder were documented in the research logbook.

The fourth stage focused on reinforcing the intended messages conveyed by participants through their photographs. This process aids the public to comprehend the context and meaning behind each photograph, as interpreted by the participants. Furthermore, this interpretation facilitated the creation of a photo booklet for distribution within the community during exhibitions.

All stages of the research were conducted between July and December 2022, with data collection processes carried out by experienced research assistants with the support of the researcher team.

### Data analysis

A thematic analysis was conducted using an interpretive phenomenological approach to explore the understanding of participants experiences of exclusion etc., focusing on the meanings derived from specific experiences, events and conditions [37]. The first author (AK) led the data analysis process, with coding on each transcript performed collaboratively by the first and second authors (AK and WW). Any disagreement regarding coding was resolved through discussions with AK, WW, MB, and MH. The first author (AK) then created a descriptive table detailing each photo and its associated story and arranged a caption based on the narrative. The research team further discussed the findings and narrowed the themes into five with several categories. Quotations were selected to illustrate the themes and categories obtained from the interview results, which were translated into English.

### Ethical considerations

Ethical approval for this investigation was granted by the Research Ethics Commission—University of 'Aisyiyah Bandung (252/KEP.01/UNISA-BANDUNG/X/2022). The participants received informed consent to be read at the first meeting. Furthermore, the first author (AK) explained the research procedures, objectives, and the required time commitment. Written informed consent was obtained from participants, covering their participation from the workshop stage to photo taking and focus group discussions. The participants also provide written consent to share photos for publication, booklets, and photo exhibitions.

## Results

Eighteen participants, including four MSM, five TG, four PID, and five FSW, participated in the photovoice. Among these 18individuals, 67% were HIV positive, with a mean age of 37.3 years (SD: + 8.7). Most key populations were high school graduates (72%), self-employed (56%), and lived independently (44%). Most participants (61.1%) experienced social exclusion from the community and a detailed characteristics is shown in Table 1.

**Table 1:** Characteristics of participants

	**Community**	**Age**	**HIV status**	**Education**	**Occupation**	**Place to live**	**Number of photos**	**Exclusionary treatment**
KP 1	MSM 1	34	+	Diploma	Private employee	Parents' house	4	Peers
KP 2	MSM 2	28	+	Senior high school	Freelance	Parents' house	4	Family dan healthcare facilities
KP 3	MSM 3	35	+	Senior high school	Self-employed	Parents' house	5	Family and peers
KP 4	MSM 4	27	+	Junior high school	Self-employed	Parents' house	5	Peers
KP 5	TG 1	33	+	Senior high school	Singer	Parents' house	4	Family dan healthcare facilities
KP 6	TG 2	60	+	Senior high school	Self-employed	Own house	5	Public community and healthcare facilities
KP 7	TG 3	44	+	Senior high school	Self-employed	Own house	2	Public community and workplace
KP 8	TG 4	28	+	Senior high school	Self-employed	Rented house	5	Public community and government apparatus
KP 9	TG 5	27	+	Junior high school	Self-employed	Parents' house	4	Government apparatus
KP 10	PID 1	40	-	Senior high school	Self-employed	Parents' house	3	Family and public community
KP 11	PID 2	42	-	Diploma	Self-employed	Parents' house	2	Family and public community
KP 12	PID 3	40	-	Senior high school	Private employee	Own house	3	Public community
KP 13	PID 4	40	+	Senior high school	Private employee	Rented house	3	Family and public community
KP 14	FSW 1	51	-	Junior high school	Self-employed	Own house	5	Government apparatus, family, and workplace
KP 15	FSW 2	39	+	Senior high school	Housewife	Own house	4	Public community
KP 16	FSW 3	34	-	Senior high school	Self-employed	Rented house	5	Public community
KP 17	FSW 4	30	-	Senior high school	Self-employed	Own house	4	Family and public community
KP 18	FSW 5	40	+	Senior high school	Self-employed	Own house	4	Public community, healthcare facilities, and workplace

The analysis of transcripts and photographs showed four main themes conveying the experiences of key populations regarding social exclusion practices. 1) limited access like unequal treatment, disadvantage, and harassment, 2) social and spiritual impact, 3) coping mechanisms, and 4) self-reflection through photovoice (see Table 2).

**Table 2:** Data analytic framework

Themes Emerges	Topics	Classifications	Categories	Sub-categories	Code
Limited access: unequal treatment, disadvantage, and harassment	Social exclusion practices	Family	Excluded and isolated from families	Being abandoned by family	Separation of living quarters and cutlery
					Families ignored and did not look for them when they ran away
					Family does not care when they live alone
			Family rejection and harassment	Receiving judgement and harassment from family	Cursed and prayed for badly by parents
					Suffer humiliation and domestic violence
		Workplace	Limited access and difficulty in choosing a job	Difficulty in obtaining and choosing employment	Employment termination due to HIV status
					Limited choice of occupation due to education and anticipated stigma
		Public community (peers, neighbors, government apparatus, community organizations)	Identity labelling and stereotypes	Receiving judgment from the community and harassment	Considered a sinner whose repentance will not be accepted
					Seen as the rubbish of society and carriers of disease
				Being abused and physical violations	Expelled from the community while receiving a beating
		Healthcare facilities	Healthcare Disparities	Inequality of treatment, unwillingness to disclose status and identity	Complex health care procedures linked to identity
					Fear of disclosing status and identity due to distrust of health workers
Social and spiritual impact	Self isolated	Social-self exclusion	Being isolated and excluded	Feeling left out and isolated by people who are not from their community	Feel unfamiliar and afraid to gather with people who are not their community
		Spiritual-self exclusion	Spiritual struggle	Doubting God's forgiveness	Feel themselves sinful and unworthy of forgiveness
					Felt that his/her prayers would not be accepted by God
Coping mechanisms	Self management	Personal capabilities and positive beliefs	Self-Sufficiency and independence	Struggling for life	Do all the work to live independently and not depend on others
					Trying to be ARV adherent to live a longer and healthier life
		Social support	Empowerment and social acceptance	Build motivation and self-image to be accepted in society	Attempting to proof of self-capability with achievements
				Peer group support as motivation/support needed	Find protection of peers in their community
Self-reflection through photovoice	Self-awareness	Self-empowerment	Empowerment through photovoice	Photovoice as expression	Feel free to express themself through photos
					Help them to be more confident to interact with the community
			Self-Reflection and emotional understanding	Help them to building self-awareness and guilt	Help them flashback to the past and realize their own mistakes

### Limited access: unequal treatment, disadvantage, and harassment

#### Excluded and isolated from families

**Fig. 1:**
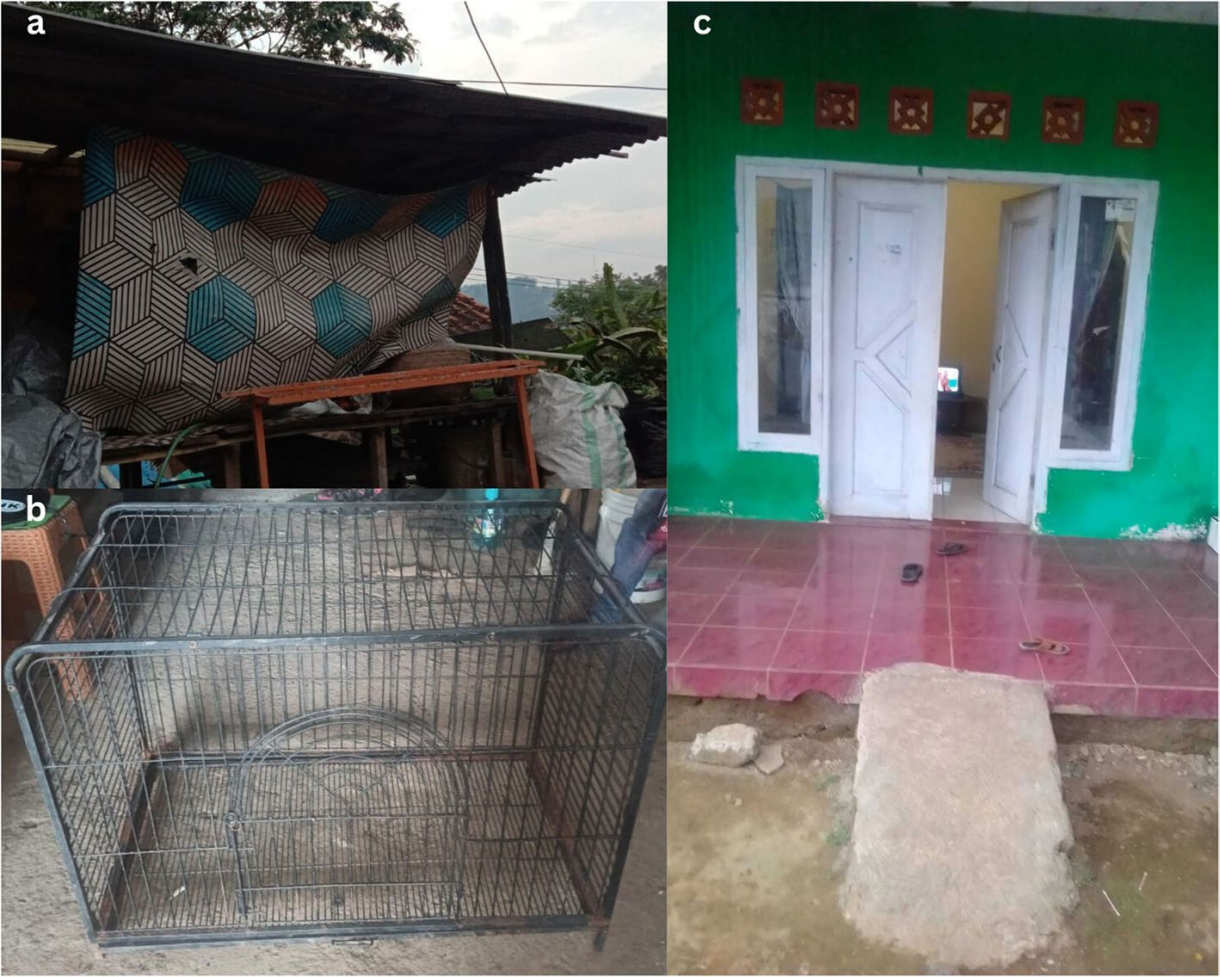
(**a**) KP5, TG1 Why was I left in a hut of seclusion? (**b**) KP2, MSM2 I feel imprisoned in an iron cage!, (**c**) KP17, FSW4 When will that door be opened for me?”

All participants described encountering various social exclusion practices within their family, workplace, community, neighbourhood, and healthcare facilities. These exclusion practices were more pronounced for those who disclosed their HIV status, who described experiencing severe consequences, including family estrangement environment, forced eviction, neglect, social isolation, abandonment, and, in some cases, even considered burdensome, and looming threats of imprisonment. Through emotional photo illustrations, these exclusionary practices were conveyed through powerful narratives and messages, such as Why was I left in a hut of seclusion? When will that door be opened for me? and I feel imprisoned in an iron cage! These messages were aimed at the broader community, especially families, which was originally intended as an important source of support for survival, as shown in Fig. 1.

One of the participants, a transgender woman, captured an image of a makeshift house that had previously been their residence. This photo was taken at the time of disclosing their HIV status, with the hope of receiving support but they ended up being degraded, ostracized and abandoned.

*"…this was my hut, after my family discovered I had HIV, they confined me to this makeshift shelter and exiled… hey eat! That was the way I was addressed during mealtime, not at the dining table, but kept in front of the door like I was a pet… how degrading was that?” (*Fig. 1(a)*; KP5, TG1, 33 years old).*

Regarded as a burden and causing shame, another participant, a male, recounts being threatened by family and restriction and surveillance imposed after their disclosure.

*"… I was threatened by my family, even to the point of contacting the police to be jailed… I tried to explain the reasons behind my HIV status, but they refused to listen. As a result, my life has been restricted ever since… movements, social interactions, and outings all need to be reported…” (*Fig. 1(b)*; KP2, MSM2, 28 years old).*

Following the birth of the child, a female sex worker (FSW) was abandoned by family due to the FSW occupation. Even to this day, the family refuses to accept the FSW, as indicated in the following excerpt.

*"… My parents have rejected me because I had a child from my workplace… the identity of the father is unclear… despite my desire to lead a normal life like everyone else… to be able to socialize with other children and families…" (*Fig. 1(c)*; KP17, FSW4, 30 years old).*

#### Difficulty in obtaining and choosing employment

**Fig. 2:**
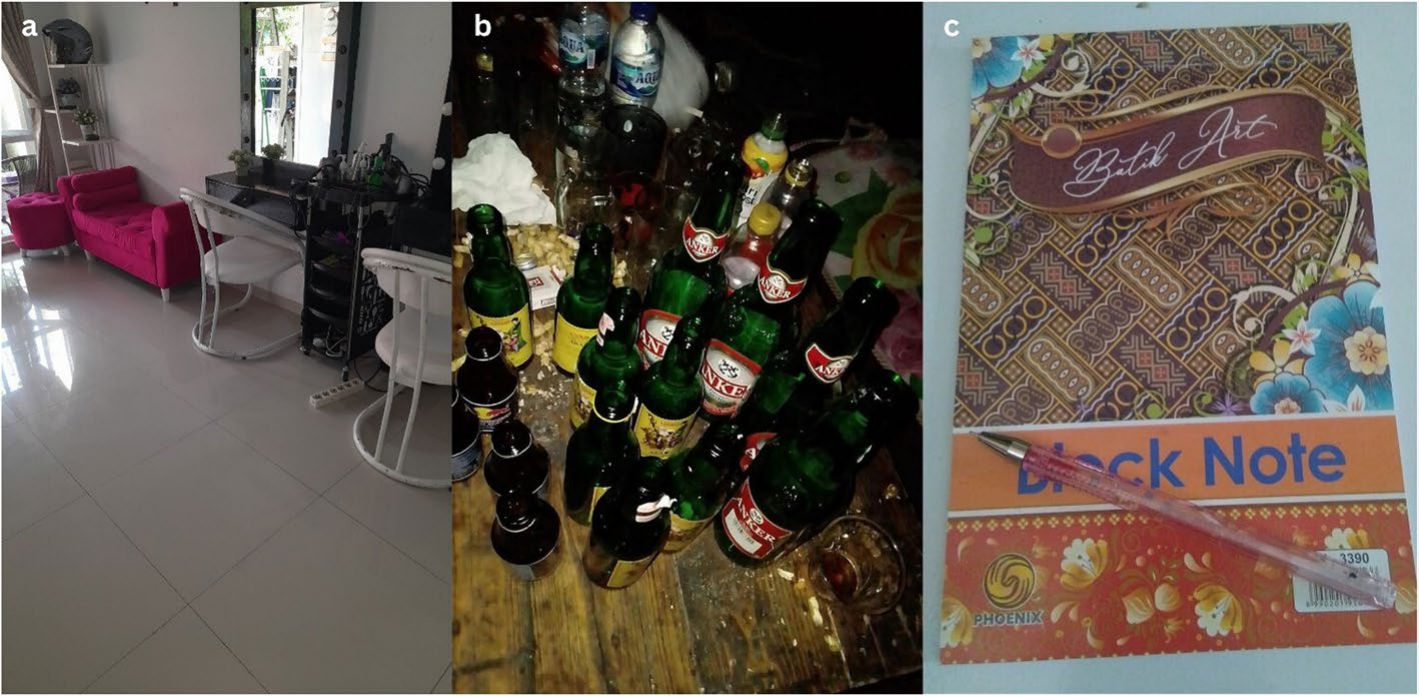
(**a**) KP7, TG3 this is my only workplace, (**b**) KP14, FSW1 this bottle is my companion while working, I have no other choice! (**c**) KP18, FSW5 my dream of working in the laboratory and carrying out research activities shattered!”

As a result of societal and workplace exclusion, employment opportunities and choices are restricted, leaving members of some key populations with little choice but to work independently, such as in salons, stage performers, and sex workers. Participants photos depict sentiments like “this place is my only workplace”, “this bottle is my companion while working, I have no other choice!" and “my dream of working with books shattered!” conveying their feelings of limited access to employment. Transgender women spoke particularly of limited job opportunities due to being ridiculed by those around them. Others describe having to work as a sex worker due to low education and the need to support their families, despite a deep desire to escape from this occupation. One participant recount turning to sex work after being dismissed from the previous job on disclosing the HIV status, as shown in Fig. 2.

*“Individuals are of the opinion that: …men are supposed to engage in manual labor, why do they have to work in a salon? …what else can we do? There are not many job options for transgenders. We also need money to survive, is it wrong to engage in such job?” (*Fig. 2(a)*; KP7, TG3, 44 years old).*

*“I cannot resign from this job because I am the breadwinner, even though it is an inappropriate job… I must live in the localization… because I have six children who must be feed!” (*Fig. 2(b)*; KP14, FSW1, 51 years old).*

*“Before I became B20, I was a teacher at a kindergarten. After being diagnosed with HIV, the foundation suddenly dismissed me unilaterally without giving a clear reason… I was just asked to quit…” (*Fig. 2(c)*; KP18, FSW5, 40 years old).*

#### Receiving judgment from the community and harassment

In the community, key populations frequently face restrictions and violations of their rights, experiencing exclusion from the social environment due to behaviors that deviate from societal norms, religious beliefs, and cultural expectations. Despite claiming they have never harmed anyone in their lives, these individuals also realize that their behavior is unjustified.

**Fig. 3:**
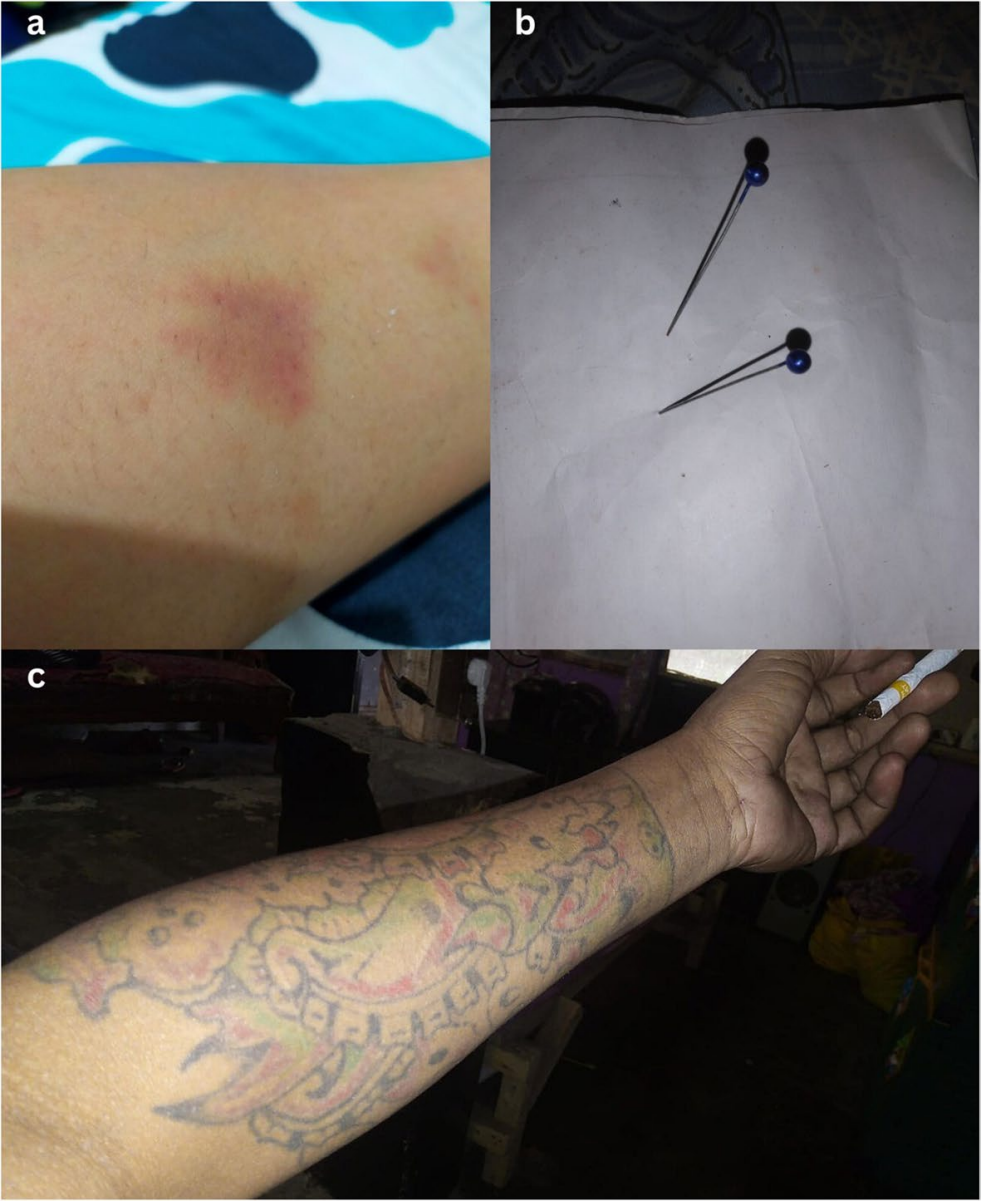
(**a**) KP8, TG4 this bruise shows the extent of their cruelty! (**b**) KP3, MSM3 Their words hurt more than needle pricks, (**c**) KP12, PID3 this tattoo makes them go away

Through photos, illustrations ‘like this bruise show the extent of their cruelty! was conveyed. Their words hurt more than needle pricks, and this tattoo makes them go away, as shown in Fig. 3.

The rights violations were daily transgressions that included violence, with instances of being verbally bullied, stereotyped, and underestimated, and physical violence. A transgender woman in this study highlights this community-enacted violence, questioning why the community relied on physical violence as a means to exclude them.

*“On that day, I was with a colleague, waiting for a customer by the side of the road, on the edge of a rice field… suddenly, a group of community organizations approached us, what transpired was something I had never imagined. We were beaten with wood, kicked, and pushed into a rice field… I just wanted to make a living, assuming this is wrong, why not educate us properly, rather than using violence?” (*Fig. 3(a)*; KP8, TG4, 28 years old).*

These rights violations meant that key populations frequently endured judgments that surpassed the limits of their humanity – they inflicted emotional harm – sometimes from those closest to them. These stigmatizing, discriminatory and exclusion practices created pain, with one participant describing the emotional pain of these practices leading to the initiation of injecting drug use, which further excluded them from family and friends.

*“I considered him a friend until I dared to reveal my condition, but his comments were heart-piercing. He said I would be the fuse of hell…there would be no chance for repentance because the sins committed were too much… Since then, I no longer considered him a friend.” (*Fig. 3(b)*; KP3, MSM3, 35 years old).*

*“… Since someone introduced me to needles, I became acquainted with tattoos and eventually developed an addiction…. friends, neighbors, and even my parents distanced themselves… My family kicked me out, and I have not seen them till lately…” (*Fig. 3(c)*; KP12, PID3, 40 years old).*

#### Inequality of treatment, reluctance to disclose status and identity

**Fig. 4:**
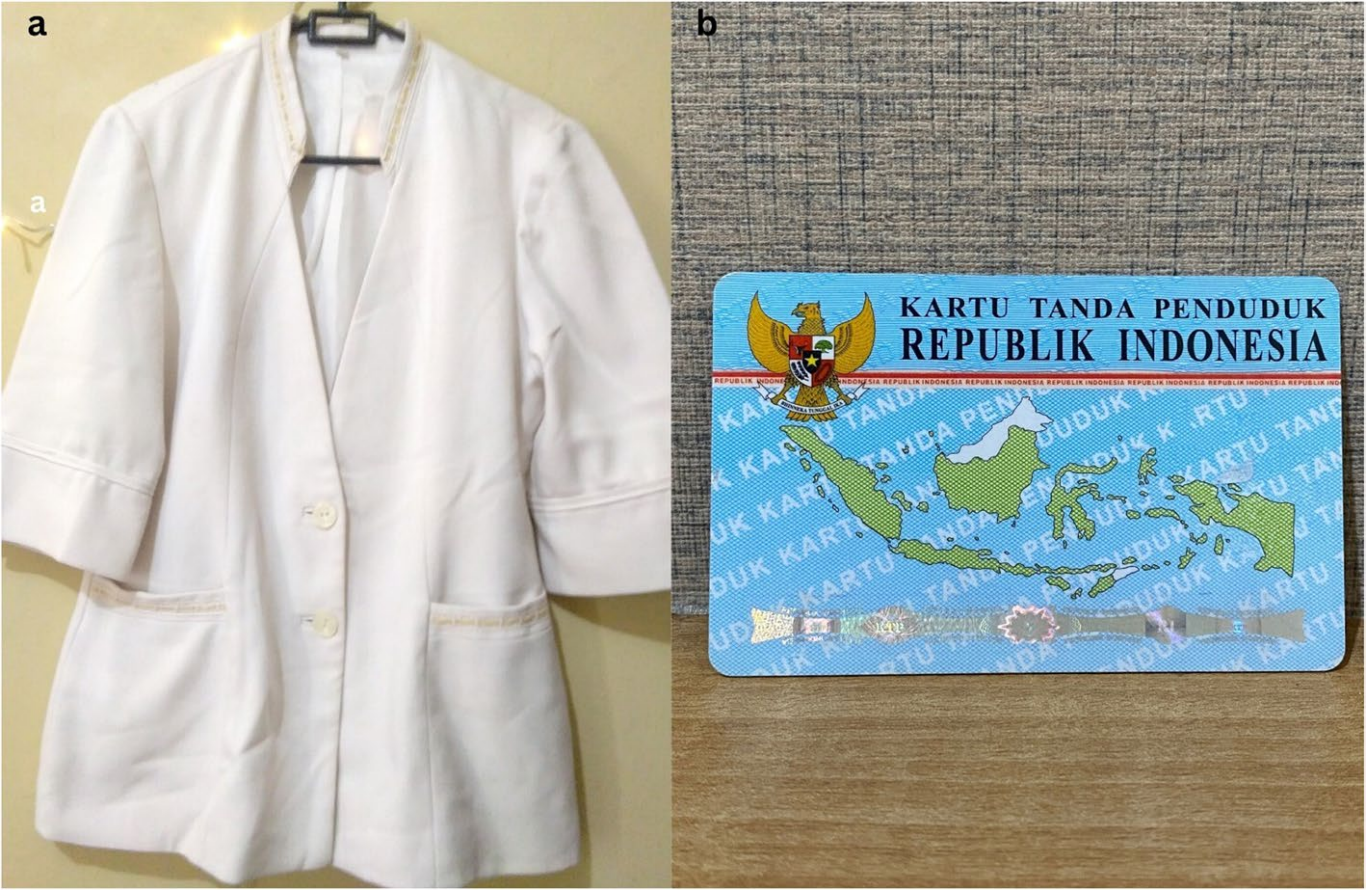
(**a**) KP18, FSW5 expressed fear that the symbolic white coat may be stained with their blood. (**b**) KP6, TG2 There are only two identities on this card!

Ensuring that health services are staffed by individuals who understand and provide exceptional care is essential to addressing disparities experienced by various key populations. In addition, transgender individuals face unique challenges that hinder their access to suitable and appropriate healthcare. Identity issues contribute to their reluctance to seek care in places that ideally cater to all, regardless of status and identity. Despite the expectation that disclosing their status would lead to personalized and understanding care, for transgender woman the reality is often different and complex. The available healthcare services are complicated and differentiated. This complexity was conveyed through photos of white coats and residence cards as a symbol of inequality in health services, as shown in Fig. 4.

*“I came to the health center to receive the COVID-19 vaccine… During consultation, the doctor asked about the medications I was taking, including ARVs, I provided the necessary information… However, to my surprise, the doctor refused to administer the vaccine despite been referred from the hospital. The next day, assistance was gotten from a different place and finally received the necessary service. The significant difference in the quality-of-service prompts questions about the inconsistency in health care, like why the service was different yesterday.” (*Fig. 4(a)*; KP18, FSW5, 40 years old).*

*“All identity-related matters are often confusing… when seeking treatment in the hospital, I am consistently asked about my gender status, even though my appearance includes long hair, wearing bangs, makeup, and skirts … they still question whether I am a man or a woman.” (*Fig. 4(b)*; KP6, TG2, 60 years old).*

### Social and spiritual impact

#### Feeling left out and isolated by individuals who are not from their community

It is understandable that in the face of ongoing stigma, discrimination and exclusion, some key populations eventually self-isolate, driven by fear of interacting with those people both inside and outside their community. This self-imposed withdrawal can lead to feelings of inferiority and alienation in the broader social environment, where the prevalent fears of this exclusion hinder meaningful social interactions and integrations.

*“…I am tired of living in the vibrant localization, especially the nightlife area… I desire to return to a more peaceful existence within the community…” (KP14, FSW1, 51 years old).*

**Fig. 5:**
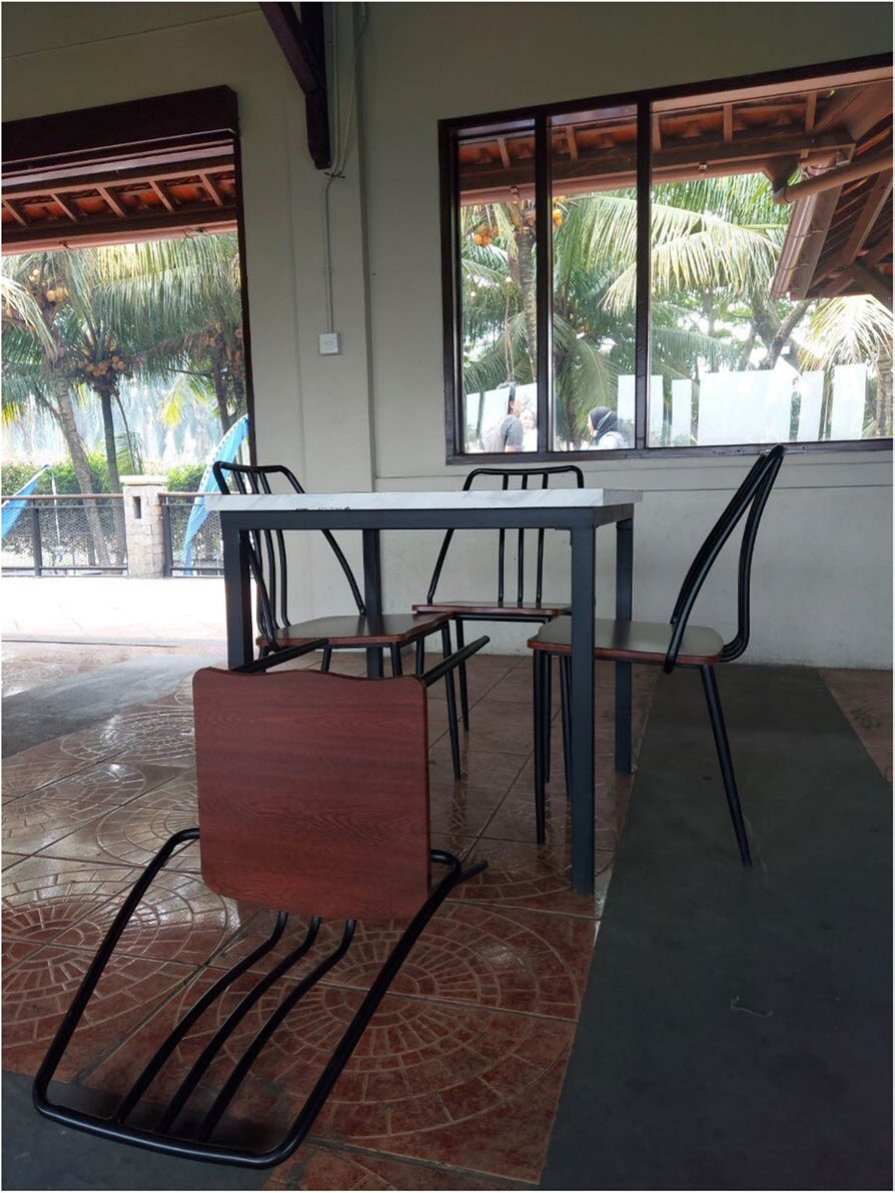
KP1, MSM1 The chair is different, just like being left out

Several participants decided to detach themselves from the community, even from other key populations. They considered themselves dirty and unworthy of being in the community, and for fear of being neglected, they isolated themselves within the localization. One MSM described feelings of inferiority and exclusion from the community by using an upturned chair as a symbolic representation, as shown in Fig. 5.

“*I often felt inferior and excluded… one day I decided to overcome this by attempting to mingle with others, unfortunately, my sincere efforts to connect… were met with harsh treatment and harassment…” (KP1, MSM1, 34 years old).*

#### Doubting God's forgiveness

**Fig. 6:**
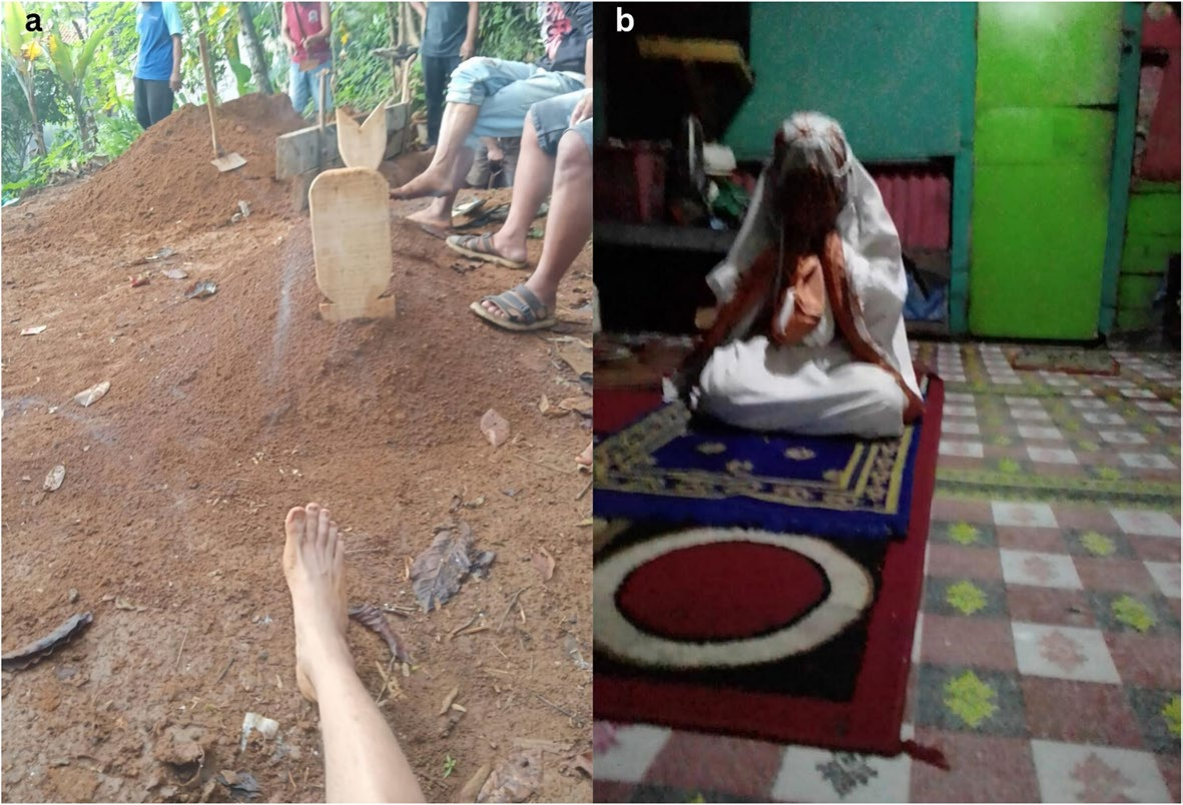
(**a**) KP4, MSM4 Sooner or later I'll be one with the ground, (**b**) KP14, FSW1 Will God accept my apology?

Intimidation and rejection from families and neighbors, was also prominent in the stories of key populations, stories that frequently contained the experience of depression and regret. This emotional toll extended beyond the social the spiritual, fostering feelings of self-doubt. Some participants blamed themselves for the situation, leading to a resigned anticipation of death, as shown in Fig. 6a. In addition to social isolation, they also felt spiritual self-exclusion and hesitated to ask for forgiveness because they were uncertain of the ability of God to forgive. For example, an FSW describes here:

*“I wish to find my way back to the path that brings contentment... but I am unsure whether God would forgive me...” (KP14, FSW1, 51 years old)*

The emotions summarized in phrases like I am despicable, I do not deserve to be among them, and Will God accept my sinful repentance? were clearly depicted in the shared photos, as shown in Fig. 6b. The photos of graveyards and worship services served as symbolic representations, allowing them to express feelings of regret and uncertainty regarding God's forgiveness as shown in Fig. 6.

*“Often, I struggle with self regrets and blame myself for the situation… a relentless fear of potentially worsening conditions due to other diseases becomes all-consuming. I know regret does not change anything… sooner or later, we will die…” (*Fig. 6(a)*; KP4, MSM4, 27 years old).*

*“I am overwhelmed by shame… feeling unworthy to prostrate before Allah… I question whether there is still forgiveness for me, considering the countless nature of my sins (participant sobbed)…” (*Fig. 6(b)*; KP14, FSW1, 51 years old).*

### Coping mechanism

#### Struggling for life

The key populations frequently encountered mistreatment, ostracism, and abandonment. Despite facing these challenges, participants showed resilience by actively managing their lives and striving to survive. Describing instances where they were trying reduce what they call a burden on their families and neighbours, by living independently and taking on any available work, as highlighted by the quote below.

*"…I do whatever it takes to make money, and feed myself without causing trouble for others… I find different ways to survive… such as slaughtering and consuming halal pets like snakes, as long as I do not steal…” (KP10, PID1, 40 years old).*

The need to provide for family required that participants work in areas and sectors that they may otherwise not want to engage with. Described by a FSW as being the breadwinner of the family, they had no choice but to continue working in an occupation they didn’t want to do because there are no other sustainable employment alternatives.

*“…I feel compelled to stay in this localization because, as the breadwinner for my six children, working is a necessity for our survival. Although, this work is also against my conscience…" (KP14, FSW1, 51 years old).*

Some participants who were living with HIV, described making concerted efforts to survive by diligently taking ARVs, even in the face of ongoing stigma and discrimination in the health service. Their conviction lies in the belief that consistent ARV use preserves their health and acts as a preventive measure against other diseases, thereby keeping them alive.

*“I try to seek medical treatment because it is the only way to stay healthy and add meaning to my life. Pursuing treatment is essential to avoid other diseases, motivating me to maintain a positive outlook!" (KP4, MSM4, 27 years old).*

#### Build motivation and self-image to be accepted in society

All participants shared a consensus that relying on others or even family for an improvement in their quality of life was not an option. Instead, they tried to maintain a positive outlook and find solace in focusing on their work and career despite the physical and mental hardships encountered. Despite the pain, their collective aspiration to participate in and reintegrate into society was described by some positively – particularly peer-based activities such as motivating and inspiring others, as well as sharing experiences and providing education to prevent individuals from falling into similar challenging circumstances.

*“….We actively engage in community activities, striving to be a positive beacon. We try to engage in positive activities, thereby contributing to the happiness of the community, fostering since of joy in the process…” (KP4, MSM4, 27 years old).*

Some participants worked hard to prove to their families and society at large that, despite their pain, they had the ability to be productive and achieve success.

*“…we make it clear to the community and our families that we are no different from anyone else… we are capable, creative and skilled, showcasing our proficiency in various activities…” (KP15, FSW2, 39 years old).*

*“…transgender individuals always encounter negative stereotypes due to their appearance. However, through our work, we aim to show our abilities to the community we aim to gradually break down social barriers and actively engage in community activities… For example, I was invited to do makeup during the carnival, providing an opportunity to connect, participate, and socialize effectively within the community." (KP6, TG2, 60 years old).*

Even when capable of independent living, these individuals still need support to lead fulfilling lives. In addition to managing stress and maintaining health, they also need support in accessing employment and health services. This essential aid extends not only from families but also from peers, communities, and health workers.

*“…we need support rather than exclusion because those of us who are already B20 will continue to take HIV medication, and our families, acting as the frontline, will continue to be constant reminders.” (KP5, TG1, 33 years old).*

*“(for health workers)… let us not focus on caste distinctions, we are the same, I am human…they too have experienced pain, and we need each other, therefore, there is no need for discrimination.” (KP2, MSM2, 28 years old).*

*“From this peer support group, I learned to respect each other and to humanize individuals, regardless of their gender identity, sexual orientation, or any other aspect… We support and care for one another, ensuring the well-being and quality of our lives.” (KP13, PID4, 40 years old).*

### Self reflection through photovoice

All participants stated that photovoice serves as a medium to express their disappointment, which they are unable to convey in words, as well as bringing about a sense of relief—like releasing burdens that were initially difficult to convey verbally. Furthermore, photovoice also helped some participants to be more confident, motivating them to participate and coexist actively within the community. A particular transgender woman, described how photovoice became a powerful outlet for emotional expression and a catalyst for newfound confidence in social interactions. They explained:

*“The photos I took were indeed a summary of my personal experiences, reflecting a journey that was difficult to convey directly in words, through these photos and activities, I discovered a liberating outlet where I could openly share my heart without the fear of being rejected and bullied. The process not only increased my confidence but also reignited my motivation to participate socially… It has truly been an enjoyable experience!" (KP8, TG4, 28 years old).*

Another participant described how photovoice increased their optimism, and was reassured of the love of God:

*“I have become more confident… and less pessimistic. Realizing that many individuals are facing greater challenges than mine… it turns out that God has not abandoned me…” (KP14, FSW1, 51 years old).*

Apart from enhancing their confidence, the participants stated that engaging in photovoice allows them to cultivate a greater sense of self-appreciation. Another transgender woman shared the struggles with physical and psychological pain during this research, stating how this process contributed to a profound appreciation of herself.

*"(through this project) … a little flashback, I have been able to appreciate my struggles starting from not being accepted by my family to being abandoned, to the point where I have managed to rise and live like a normal person … the positive impact of this project has revealed that every detail of our actions holds immense value.” (KP5, TG1, 33 years old).*

## Discussion

In this research, a photovoice was used to explore and capture the authentic and unique narratives based on non-verbal and verbal stories of key population’s everyday lives [21].

These stories were overwhelmingly concerned with unfair treatment, stigma, discrimination and social exclusion practices encountered within the family, community, and health service environment and the significant impacts that these practices had on their lives, but also included stories of strength and resistance. The conditions that lead to social exclusion had a significant effect on the lives of the individuals, and the stress associated with these situations was discussed. In addition, the participants’ self-reflection was facilitated through photovoice.

### Views of social groups' values, beliefs, and norms towards key populations

The results of this research showed that the living conditions, experiences, and challenges of the participants circulated around their lives as key populations. These individuals endured social exclusion, stigma and discrimination, which at times was what is referred to double or multiple-levels discrimination related to their gender identity, sexual behavior and HIV status [38]. Participants were often subjected to judgement, violence and discrimination from various social groups such as family, friends, the community, and health workers, which was compounded by suffering, abandonment and lack of support.

Polarized perspectives of values, beliefs and norms within various social groups significantly influence the social exclusion experiences of this marginalized group. Deeply entrenched conservative norms within society foster negative attitudes and behaviors towards key populations, viewing them as abnormal, posing threats to societal values and morals, and deviating from the norm, justifying rejection [39–41]. These societal values, beliefs and norms are often filtered through the perspective of social groups, neglecting the individual experiences of key populations and creating an environment of prejudice, negative judgement and unfair treatment [42–44]. Men who have sex with men (MSM) in Indonesia are seen as sexual deviants and contaminate the culture. Those who are considered sexually deviant tend to be regarded as someone who is sick (has mental problems), sinful, and even considered not an excellent Indonesian citizen [45, 46]. Similarly, transgender people are seen as sexually deviant, contaminated, and often rejected by most Indonesian cultures [15, 47].

In Indonesia, stigma and discrimination against key populations is evident with beliefs from these stigmatising practices resulting in self-stigma—the majority of key populations contend with the belief that they lead sinful lives and do not belong in society [12]. This internalized stigma is a direct result of community prejudice, where the assumption is that the pain they experience is a consequence of perceived immoral mistakes and deviant behavior, equated with sins committed [10, 48, 49].

This stigma and discrimination are particularly significant in religious life in Indonesia. One area that is recently receiving attention in this space is the importance of religious leader participation in the provision of education to increase public understanding and helping to resolve conflicts between religious scripture and health promotion, however, in Indonesia this space is characterised by absence, or lack of involvement [14]. Social exclusion of key populations is also found in several countries. Several studies have shown that the inclusivity of religious leaders in Kenya can be improved through sensitivity training on GBMSM (Gay, Bisexual, Men who have sex with men) issues [50], while in Uganda the involvement of religious leaders contributed 70% to the HIV prevention strategy [51].

Health workers, expected to provide support and care, contribute to the stigmatization of key populations, their families and communities. As a result, key populations in this study and elsewhere describe barriers to health services that limit access to health services. These barriers can include psychological and social factors that become significant in the interaction between key populations and health care workers, leading to reluctance to seek care or delaying necessary visits [52–55]. This is particularly concerning as key populations are vulnerable and need regular check-ups, prompt diagnosis, and timely treatment.

The media also influences public perceptions and attitudes towards key populations, but unfortunately, it often aggravates and perpetuates stigma and discrimination due to the portrayal of key populations as engaging in deviant behavior and not conform to the prevailing cultural norms in society [17]. Recognizing the impact of communication and media platforms, it is crucial to address stigma through campaigns that promote accurate and positive portrayals of key populations, thereby generating empathy and cultivating a more inclusive and supportive society [18].

The results of this study focus on the importance of engaging social groups and health workers in establishing inclusive environments through storytelling based on genuine experiences. These inclusive environments empower excluded individuals, allowing them to use their voices to achieve sustainable social change, findings reported elsewhere [20, 23–25, 30, 33].

### Dealing with stress: coping strategies of key populations

Social exclusion, stigma and discrimination faced by key populations about HIV significantly affect their mental health and well-being [56, 57]. Despite these challenges, most participants showed resilience drawing on their inner resources as coping mechanisms, which were used to bounce back and recover from the sometimes-everyday experience of stigma and discrimination, effectively overcoming adversity. This resilience-building process included fostering positive social relationships and adhering to care and treatment [58].

Resilience within key populations, originates from the individuals need to survive [59]. In this study, most participants prefer developing their potential by showing their achievements and seeking community support through empowerment and active participation in public activities [60, 61]. This portrays their presence and contribution to society, empowering them individually and fostering a more inclusive neighborhood within a broader community [62].

Their success influences the ability of key populations to live a positive life by managing their internal and external resources. Internal resources such as hope, optimism, and resilience become their strategies for managing and coping with living with HIV. External resources such as support from family, friends, health workers and a sense of responsibility they have towards their families become effective strategies to cope with the impact of HIV and maintain their physical and mental health [63, 64].

Social support from peers in the same community was critical to participants in this study, particularly with respect to building the resilience and confidence of key populations [65, 66]. Like research reported elsewhere, in this study the community is thought to provide a sense of belonging and understanding, which serves as a safe place for these individuals to freely express themselves and seek emotional support [67, 68]. However, these results contradict other research where participants did not always receive social support from friends, nor see support from friends as a key factor in coping with stigma. This could be attributed to anticipated stigma and tenuous social relationships from the onset [22].

The direct participation of key populations is crucial for creating a positive community image, fostering inclusivity, and contributing to better social change [69]. The use of education and advocacy activities to empower these individuals facilitates meaningful interaction with the community [70].

### Building self-awareness: reflectivity of key populations through photovoice

Photovoice plays a crucial role in helping key populations develop self-awareness through critical reflection, serving therapeutic function by increasing self-confidence, self-esteem, and enabling free self-expression [22, 66, 71]. The photovoice method in this study allowed for individuals to freely express themselves and reflect on their values, motivations, beliefs and past experiences [72]. Participants believe this process promotes a change in attitude and facilitates a better understanding of themselves and the surrounding world [73].

Photovoice has been proven to be a valuable tool, mainly used in socially marginalized populations in various scenarios aiming to empower [24, 30, 33, 74, 75], explore experiences [20, 21, 25, 76], and capture important messages about their lives and communities [21, 26, 77]. Recognized for its effectiveness, it provides an opportunity to promote self-awareness and empowerment among key populations [78]. In addition, photovoice serves as a therapeutic medium, aiding in the personal development of key populations to overcome stigma and social barriers [66, 72].

This research results provide valuable information on how identifying the needs of key populations helps overcome social exclusion and stigma through coping mechanisms and self-reflection. Furthermore, this research also serves as a medium to promote positive portrayals of key populations, fostering empathy and contributing to a more inclusive and supportive society [18]. The results also bridge key populations facing challenges in verbally expressing their experiences, evoking a humane response from individuals through photos based on their true-life stories.

### Strengths and limitations

This study has several limitations, such as the involvement of a small number of participants from each group, restricting the generalization of our research to everyone in key population community’s. Using snowball sampling in recruiting PIDs also meant that participants were recruited from the same networks. This may have led to an incomplete picture of their experiences of social exclusion in the community.

However, the present research visually captures the real-life experiences of key populations, not only with HIV-related social exclusion but also their specific behaviors, which face societal norms and cultural and belief-based unacceptance. In Indonesia, this research focused on a cultural context with distinct beliefs and norms regarding key populations compared to developed or socially accepted nations. Therefore, future research should further compare countries with diverse characteristics to explore the differences and limitations of the various situations. This approach would include adjusting the specific values, norms and beliefs in each society under consideration.

## Conclusions

This research highlights the importance of participants' openness in sharing their experiences, generating valuable findings that can shape HIV policy and practice. These findings have great potential to strengthen approaches that promote the acceptance and inclusion of key populations in society.

Through the photovoice medium, key populations could convey important messages, providing an effective strategy for building empathy within the community. The implications of the findings are highly relevant for designing HIV policies and practices with a focus on inclusion and acceptance and demonstrate that the knowledge generated from this research can be a foundation for positive change in society.

We advocate making the photovoice efforts accessible to a wider audience through exhibitions and various media. This is expected to expand the positive influence of photovoice as a medium to convey important messages from participants. It is a strategy to create a more inclusive environment and encourage the public to support key populations in their fight against HIV.

## Data Availability

The data that support the findings in this research are available in a safe place by the authors (AK and WW). We cannot open the data to public access to maintain the confidentiality of key populations.
